# Periosteum-derived mesenchymal stem cell alleviates renal fibrosis through mTOR-mediated Treg differentiation

**DOI:** 10.1080/0886022X.2023.2212079

**Published:** 2023-05-23

**Authors:** Yongsheng Luo, Kuanxin Zhang, Jiaheng Wu, Hao Zeng, Juntao Chen, Pingbao Zhang, Jingjing Guo, Cuidi Xu, Xinhao Niu, Yin Celeste Cheuk, Shihao Xu, Yirui Cao, Yufeng Zhao, Dong Zhu, Xuanchuan Wang, Ruiming Rong

**Affiliations:** a Department of Urology, Zhongshan Hospital, Fudan University, Shanghai, China; b Shanghai Key Laboratory of Organ Transplantation, Shanghai, China; c Department of Colorectal Surgery, The First Affiliated Hospital of Zhengzhou University, Zhengzhou, China; d Shanghai Medical College, Fudan University, Shanghai, China

**Keywords:** Renal fibrosis, mesenchymal stem cell, regulatory T cells, mTOR, ischemia–reperfusion, kidney transplantation

## Abstract

**Background:**

Mesenchymal stem cells (MSCs) are the hotspots of cellular therapy due to their low immunogenicity, potent immunoregulation, and unique renoprotection. The present study aimed to investigate the effects of periosteum-derived MSCs (PMSCs) in ischemia–reperfusion (IR)-mediated renal fibrosis.

**Methods:**

Using cell proliferation assay, flow cytometry, immunofluorescence, and histologic analysis, the differences in cell characteristics, immunoregulation, and renoprotection of PMSCs were compared to the bone marrow-derived MSCs (BMSCs), the most frequently studied stem cells in cellular therapy. In addition, the mechanism of PMSC renoprotection was investigated by 5′ end of the RNA transcript sequencing (SMART-seq) and mTOR knockout mice.

**Results:**

The proliferation and differentiation capabilities of PMSCs were stronger than those of BMSCs. Compared with BMSCs, the PMSCs exerted a better effect on alleviating renal fibrosis. Meanwhile, the PMSCs more effectively promote Treg differentiation. Treg exhaustion experiment indicated that Tregs exerted an important effect on inhibiting renal inflammation and acted as a critical mediator in PMSC renoprotection. Additionally, SMART-seq results implied that the PMSCs promoted Treg differentiation, possibly via the mTOR pathway. *In vivo* and *in vitro* experiments showed that PMSC inhibited mTOR phosphorylation of Treg. After mTOR knockout, the PMSCs failed to promote Treg differentiation.

**Conclusions:**

Compared with BMSCs, the PMSCs exerted stronger immunoregulation and renoprotection that was mainly attributed to PMSC promotion for Treg differentiation by inhibiting the mTOR pathway.

## 1. Introduction

Renal fibrosis is the common pathological end point of various renal diseases, including ischemia–reperfusion (IR) injury, chronic rejection, glomerulonephritis, obstructive nephropathy, etc. [1,2]. Notably, IR injury with an intensively inflammatory response is an inevitably pathologic surgical operation process such as kidney transplantation [2,3]. When the persistent inflammation during IR injury is not promptly controlled, the pathological damage in the kidney will gradually increase, eventually leading to renal fibrosis [1–3]. Unfortunately, the current pharmacological interventions have not yet achieved prospective efficacy [4]. Hence, increasing numbers of immunologists have begun to explore cell infusion therapy for renal fibrosis [4–6].

Mesenchymal stem cells (MSCs) are the most frequently studied cellular therapy method due to their unique immune regulation and low immunogenicity [5–7]. For example, increasing research has demonstrated that MSC infusion can inhibit inflammation and improve renal fibrosis by increasing immunosuppressive regulatory T cell (Treg) numbers [8–10]. MSCs obtained mainly from bone marrow, fat, umbilical cord, muscle, and other sources have different effects on various diseases [6,11]. Bone marrow-derived MSCs (BMSCs) are the most frequently studied stem cells in cellular therapy [6–8,11]. However, the differentiation ability of BMSCs varies significantly among different individuals and ethnic origins. Their directional differentiation characteristics only focus on the early culture stage, gradually losing the ability to induce differentiation during the passage. Particularly, BMSCs from elderly individuals have slow expansion speed *in vitro* and low differentiation ability. These BMSCs are prone to aging and lose differentiation and proliferation ability, which limits the extensive application of BMSCs in treating disease [12,13].

Some studies have reported that periosteum-derived MSCs (PMSCs) originated from embryonic mesoderm have multidirectional differentiation potential with rapid expansion and stable phenotype *in vitro* [14–16]. Elderly individuals even maintain strong osteogenic activity [14]. Compared to BMSCs, increasing research has shown that PMSCs possess stronger differentiation potential [15–17]. For instance, a study on promoting osteogenesis induction reported that the early osteogenesis related indicators of PMSCs was significantly higher than BMSCs [17]. Moreover, increasing research has shown that PMSCs show promising bone regeneration ability in ectopic osteogenesis and repair of extreme bone defects [14–16]. Proverbially, MSC differentiation potential is positively associated with immune regulation ability [18,19]. Therefore, compared with BMSCs, the PMSCs possibly possessed stronger immunoregulation and renoprotection [17–19].

This study explored the differences in cell characteristics, immunoregulation and renoprotection between PMSCs and BMSCs. Moreover, the mechanism of PMSC renoprotection was further investigated through sequencing technology and knockout mice. Our results showed that compared with BMSC infusion, the transfer of PMSCs exerted robust immunoregulation and renoprotection, which were mainly attributed to the PMSC-mediated Treg promotion by inhibiting the mTOR pathway. PMSCs are expected to be a new type of cellular therapy after renal IR.

## 2. Materials and methods

### 2.1. Mice

Wild-type (WT) C57BL/6 mice (6–8 weeks, male) were obtained from the Jiesijie Company (Shanghai, China). The tamoxifen-induced mammalian target of the rapamycin (mTOR) knockout mice (ER-Cre-mTOR^flox/flox^, mTOR^–/–^ mice), C57BL/6 mice were generated by crossing mTOR^flox/flox^ mice with mice expressing Cre recombinase under the control of the ER promoter (ER-Cre), which were provided by Professor Yong Zhao from the Institute of Zoology, Chinese Academy of Sciences, Beijing, China. The genotypes of mTOR^flox/flox^ mice and ER-Cre mice were demonstrated by DNA electrophoresis with the following primers: mTOR-WT, mTOR-common, mTOR-mutant, ER-Cre 1, ER-Cre 2, and ER-Cre 3 (Table S1). The genotyping of mTOR^flox/flox^ C57BL/6 mice was utilized in the previous study [20]. The genotyping ER-Cre C57BL/6 mice were constructed in a similar manner. All mice were acclimated for at least three days before the experiments and administered based on the Guidelines of Laboratory Animals. The present study was approved by the Animal Ethical Committee of Zhongshan Hospital, Fudan University, Shanghai, China.

### 2.2. Renal fibrosis model and treatment

The renal fibrosis model and MSC therapy scenario were utilized according to the previous studies [21,22]. C57BL/6 mice were divided into the following groups. (1) NC group, the abdomen was only exposed for 0.5 h, with intravenous injection of 200 μL Dulbecco’s modified Eagle medium (DMEM, Gibco, Carlsbad, CA) from day 1 to 3; (2) IR group, with normal left kidney, the renal pedicle of the right kidney was clipped with a vascular clamp for 0.5 h to induce ischemia. Subsequently, the mice were injected with 200 μL DMEM via the tail vein, respectively, on the 0 h, 24 h, and 48 h after the vascular clamp removal; (3) IR + BMSC group, IR mice were injected with 5 × 10^5^ BMSC in 200 μL DMEM, respectively, on the 0 h, 24 h, and 48 h after the vascular clamp removal; (4) IR + PMSC group, IR mice were injected with 5 × 10^5^ PMSCs in 200 μL DMEM, respectively, on the 0 h, 24 h, and 48 h after the vascular clamp removal. The endpoint of renal fibrosis was administered on the 28th day after IR.

### 2.3. Treg depletion

The anti-CD25 antibody (PC61, Biolegend, San Diego, CA) was intraperitoneally injected with 1.5 mg/kg on day 14 before operation, day 1, and day 14 after the operation. The corresponding IgG (Biolegend, San Diego, CA) was administered as a control. C57BL/6 mice were divided into four groups: IR + IgG, IR + PC61, IR + PC61 + PMSC, and IR + IgG + PMSC.

### 2.4. Cell culture and preparation

The BMSCs (Cyagen, Santa Clara, CA) from C57BL/6 mice and PMSCs (Procell, Wuhan, China) were cultured to the 6–9th passage by DMEM with 10% fetal bovine serum (FBS, Biochannel, Nanjing, China). 1 × 10^5^ MSCs/well were cultured with the 12-well plate for the growth curves. The MSCs were counted from day 1 to 3 by Countess 3 Automated Cell Counters (Invitrogen, Carlsbad, CA). To detect cell proliferation, these MSCs were incubated at a cell concentration of 2 × 10^7^ cells/mL with 10 μmol/L 5(6)-carboxyfluorescein diacetate, succinimidyl-ester (CFSE) for 0.5 h at 37 °C. The PMSCs and BMSCs were collected respectively after the three-day cell culture and the 2nd three-day cultivation, which were subsequently detected by flow cytometry.

The single-cell suspension of the spleen was prepared directly by grinding the spleen from C57BL/6 mice. To prepare kidney single cell suspension, the kidney was cut into a 2 mm tissue block, subsequently digested by collagenase type IV (STEMCELL Technologies, Vancouver, Canada) for 30 min, and further ground by a 5 mL syringe. The single-cell suspensions of the spleen and kidney were filtered via a 40 μm cell strainer before staining by flow cytometry.

To prepare the CD4^+^CD25^–^ T cells, the spleen single cell suspension was first centrifuged via lymphocyte separation medium (Absin, Shanghai, China) and subsequently sorted by Mouse CD4 Naïve T Cell Isolation Kit (Biolegend, San Diego, CA). To explore the MSC effect on Treg differentiation, the MSCs and CD4^+^CD25^–^T cells (1:10) were seeded in a 48-well plate, with Roswell Park Memorial Institute 1640 (Gibco, Carlsbad, CA), 10% FBS, 1% penicillin–streptomycin (Gibco, Carlsbad, CA), 5 μg/mL anti-CD3 (Becton, Dickinson and Company, Franklin Lakes, NJ), 2.5 μg/mL CD28 (Becton, Dickinson and Company, Franklin Lakes, NJ), 2 mmol/L l-glutamine (Gibco, Carlsbad, CA), and 50 µmol/L β-mercaptoethanol (Gibco, Carlsbad, CA) for 72 h.

### 2.5. Flow cytometry

The stem cell surface markers of the MSCs were administered using flow cytometry, which was stained with a Fixable Viability Kit (Biolegend, San Diego, CA) and Mouse MSC Analysis Kit (Cyagen, Santa Clara, CA) for 30 min according to the instructions. The CFSE-labeled MSCs were harvested on the 2nd three-day cultivation and detected by flow cytometry, with additional staining using the Fixable Viability Kit. The spleen and kidney single-cell suspensions from experimental mice were first stained via fluorophore-conjugated antibodies, including BV510 Fixable Viability Kit (Biolegend, San Diego, CA), FITC anti-CD45 (Biolegend, San Diego, CA), PerCP/Cy5.5 anti-CD4 (Biolegend, San Diego, CA), APC anti-CD25 (Biolegend, San Diego, CA) for 30 min at 4 °C, follow by PE anti-Foxp3 (Biolegend, San Diego, CA), and PE/Cy7 anti-mTOR (Invitrogen, Carlsbad, CA) for 2 h at 4 °C. The CD4^+^T cells cultured *in vitro* were stained via BV510 Fixable Viability Kit, PerCP/Cy5.5 anti-CD4, and APC anti-CD25 antibodies for 30 min at 4 °C. The CD4^+^CD25^+^CD127^–^Tregs sorted by fluorescence activated cell sorting (FACS) were stained via BV510 Fixable Viability Kit, PerCP/Cy5.5 anti-CD4, APC anti-CD25, and PE anti-CD127 (Biolegend, San Diego, CA) antibodies for 30 min at 4 °C.

### 2.6. Western blot

Equal amounts of protein extracted from the spleen of tamoxifen induced mTOR^–/–^ mice, mTOR^flox/flox^ER-Cre-negative mice, and WT mice were used for electrophoresis and subsequently transferred to polyvinylidene fluoride membrane. The mTOR antibody was diluted at 1:10,000 (Abcam, Cambridge, MA). The normalized β-actin was diluted at 1:1000 (Abcam, Cambridge, MA).

### 2.7. The osteogenic and adipogenic differentiation

When the PMSCs and BMSCs reached 70% confluence under the complete medium, osteogenic induction differentiation medium was administered every three days until crystallization of cellular calcium salts and formation of calcium nodules. Subsequently, the PMSCs and BMSCs were incubated with Alizarin Red solution (Cyagen Biosciences Inc., Santa Clara, CA) for 5 min after these cells were fixed with 4% formaldehyde. For Oil Red O staining, the adipogenic differentiation kit (Cyagen Biosciences Inc., Santa Clara, CA) was used according to the manufacturer’s instructions after the PMSCs and BMSCs reached 80–90% confluence under the complete medium. The microscope (Olympus, Tokyo, Japan) was administered for image acquisition.

### 2.8. Enzyme-linked immunosorbent assay (ELISA)

Blood samples were collected on the days 2 and 28 after IR. The serum creatinine and urea nitrogen levels were assessed using the creatinine or urea nitrogen assay kit (mlbio, Shanghai, China) according to the manufacturer’s instructions. IR kidney samples on the day 2 after operation were harvested and lysed in lysis buffer at 4 °C for 0.5 h. The levels of IL-6, IFN-γ, and IL-10 were administered using ELISA kits (mlbio, Shanghai, China) according to the manufacturer’s instructions.

### 2.9. Histologic analysis

The kidney tissues obtained from experimental mice were immediately soaked in 10% formalin, stored through paraffin embedding, and sliced into 5 μm thickness. These slices were deparaffinized and rehydrated, and subsequently stained via hematoxylin–eosin (HE), Sirius red, α-SMA, and periodic acid-Schiff (PAS). The staining of HE was scored to assess the tubular injury according to the percentage of brush tubular dilation, cast formation, border loss, and tubular necrosis: 1, ≤25%; 2, 26–50%; 3, 51–75%; 4, >75%. The positive areas of Sirius red and α-SMA were semi-quantified by ImageJ (National Institutes of Health, Bethesda, MD). All histologic analyses were assessed by two independent pathologists blinded to the groups.

### 2.10. Immunofluorescence analysis

The staining of Foxp3 in the kidney and spleen was administered by immunofluorescence. The kidney and spleen slices were stained via anti-Foxp3 (1:100, Abcam, Cambridge, UK) primary antibody (rabbit anti-mouse) at 4 °C overnight and subsequently added with FITC donkey anti-rabbit IgG (1:400, Life Technologies, Carlsbad, CA) at room temperature for 1 h, with the DAPI (Sigma, St. Louis, MO) for nuclei. Additionally, the F4/80 staining in the kidney was administered via anti-F4/80 (1:200, Cell Signaling Technology, Boston, MA) primary antibody (rabbit anti-mouse) at 4 °C overnight and subsequently added with Alexa Fluor™ 594 donkey anti-rabbit IgG (1:400, Life Technologies, Carlsbad, CA) at room temperature for 1 h, with the DAPI (Sigma, St. Louis, MO) for nuclei. The dUTP nick-end labeling (TUNEL) staining in the kidney was administered via TUNEL Assay Kit (Cell Signaling Technology, Boston, MA) according to the manufacturer’s instructions. The fluorescence microscope (Olympus, Tokyo, Japan) was administered for image analysis.

### 2.11. Statistical analysis

The IBM SPSS Statistics 21 (Armonk, NY) and GraphPad Prism 8 software (La Jolla, CA) were used for data analysis. The differences for between-group comparisons were analyzed by an independent-sample *t*-test. The differences for among-group comparisons were analyzed by the Kruskal–Wallis one-way analysis of variance and post hoc tests with all pairwise. Data were shown as the median with interquartile range (IQR) or mean ± standard deviation (SD). Bioinformatic analysis was performed using the OmicStudio tools at https://www.omicstudio.cn/tool. *p* < .05 was considered statistically significant.

## 3. Results

### 3.1. PMSCs exhibited beneficial effects on renal fibrosis

**Figure 1.**
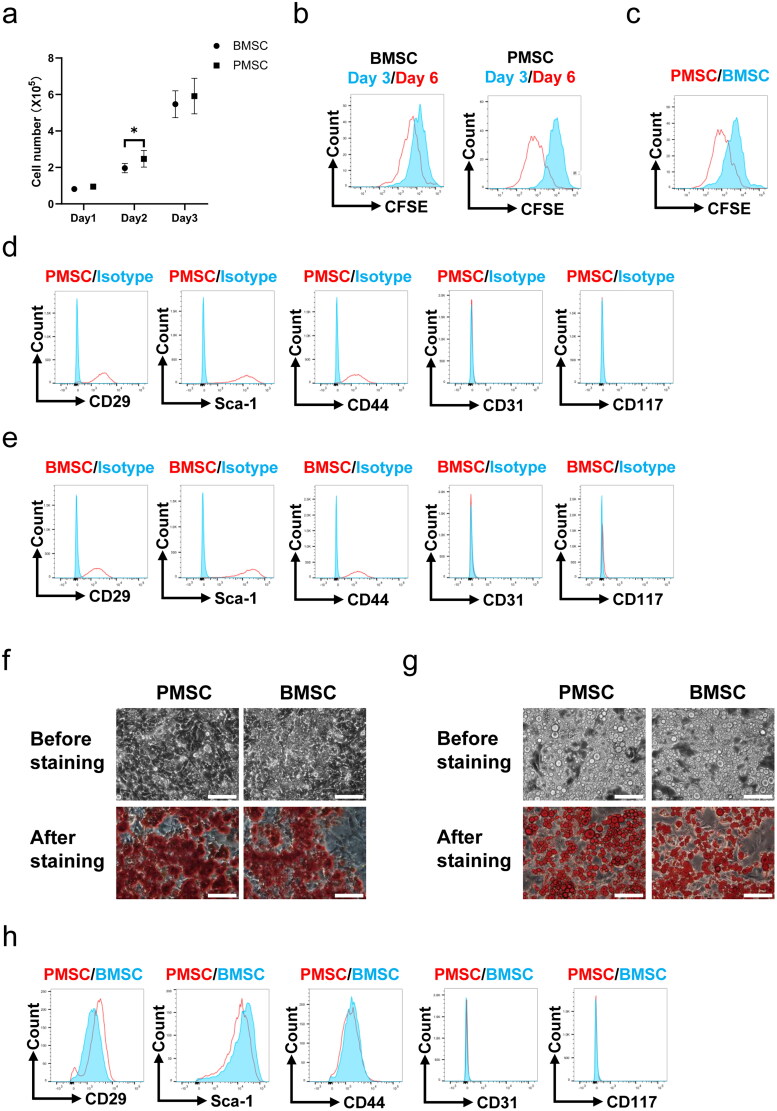
The characteristics of PMSCs and BMSCs. (a) The cell numbers of PMSCs (*n* = 6) and BMSCs (*n* = 6) during the three-day cell culture. (b) The expressions of CFSE of PMSCs and BMSCs collected after the three-day cell culture and the 2nd three-day cultivation were detected by flow cytometry. (c) The expressions of CFSE of PMSCs and BMSCs collected after the 2nd three-day cultivation were compared by using flow cytometry. (d) The expressions of Sca-1, CD29, CD44, CD31, and CD117 on the surfaces of PMSCs collected after the 2nd three-day culture were detected by flow cytometry. (e) The expressions of surface marks of BMSCs collected after the 2nd three-day culture were detected by flow cytometry. (f) The osteogenic differentiation of PMSCs and BMSCs was identified on the 21th day by Alizarin Red staining. Scale bars: 100 μm. (g) The adipogenic differentiation of PMSCs and BMSCs was identified on the 21th day by Oil Red O staining. Scale bars: 100 μm. (h) The surface marks of PMSCs and BMSCs collected after the 2nd three-day cultivation were compared by using flow cytometry. An independent-sample *t*-test evaluated differences for between-group comparisons. *p < .05.

**Figure 2.**
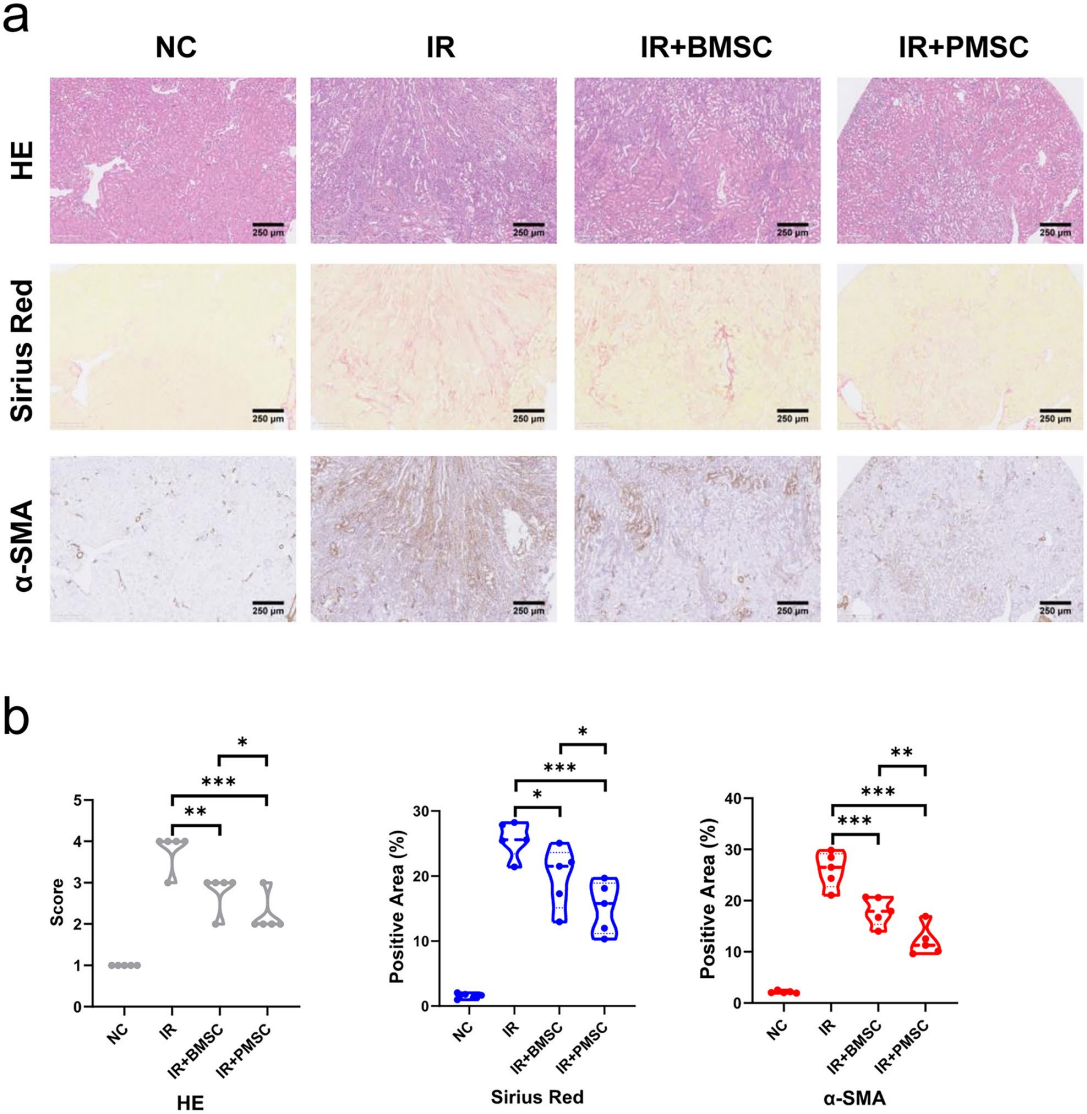
PMSCs effectively relieved renal fibrosis. (a, b) The HE, Sirius Red, and α-SMA staining were administrated to assess renal fibrosis in the NC (*n* = 5), IR (*n* = 5), IR + BMSC (*n* = 5), and IR + PMSC (*n* = 5) groups. Scale bars: 250 μm. The Kruskal–Wallis one-way analysis of variance and post hoc tests with all pairwise for among-group comparisons. *p < .05; **p < .01; ***p < .001.

During the three-day cell culture, the growth curves showed that the cell numbers of PMSCs were significantly higher than those of BMSCs (*p* < .05, Figure 1(a)) on the second day. However, no differences were observed in the cell proliferation tested by CFSE between the PMSC and BMSC groups (Figure S1) after three-day culture. Subsequently, these MSCs were passed on to the next generation and cultured for another three-day. Comparing the expressions of CFSE between the 1st and 2nd cultivation, the PMSCs exhibited more obvious proliferation than BMSCs (Figure 1(b)). Moreover, the CFSE expressions of PMSCs were less than those of BMSCs after the 2nd cultivation (Figure 1(c)). Additionally, the PMSCs (Figure 1(d)) and BMSCs (Figure 1(e)) were identified by testing the positive expressions of CD29, Sca-1, and CD44 on the surface of stem cells, as well as negative markers including CD31 and CD117. Meanwhile, Alizarin Red and Oil Red O stainings revealed that the osteogenic (Figure 1(f)) and adipogenic (Figure 1(g)) formations of PMSCs were more obvious than those of BMSCs. However, no differences were observed in surface markers between the PMSC and BMSC (Figure 1(h)) after the 2nd cultivation. To assess the PMSC and BMSC effects on renal injury, renal function and pathology were analyzed on the 28th day after IR. The serum creatinine and urea nitrogen levels showed no differences among the NC, IR, IR + BMSC, and IR + PMSC groups (all *p* > .05, Figure S2). However, the HE, Sirius Red, and α-SMA staining showed that the levels of renal fibrosis were significantly lower in the IR + PMSC group and IR + BMSC group than those in the IR group. Moreover, compared to BMSC, the PMSC infusion more effectively relieve renal fibrosis (all *p* < .05, Figure 2(a,b)).

### 3.2. PMSCs promoted Treg differentiation

**Figure 3.**
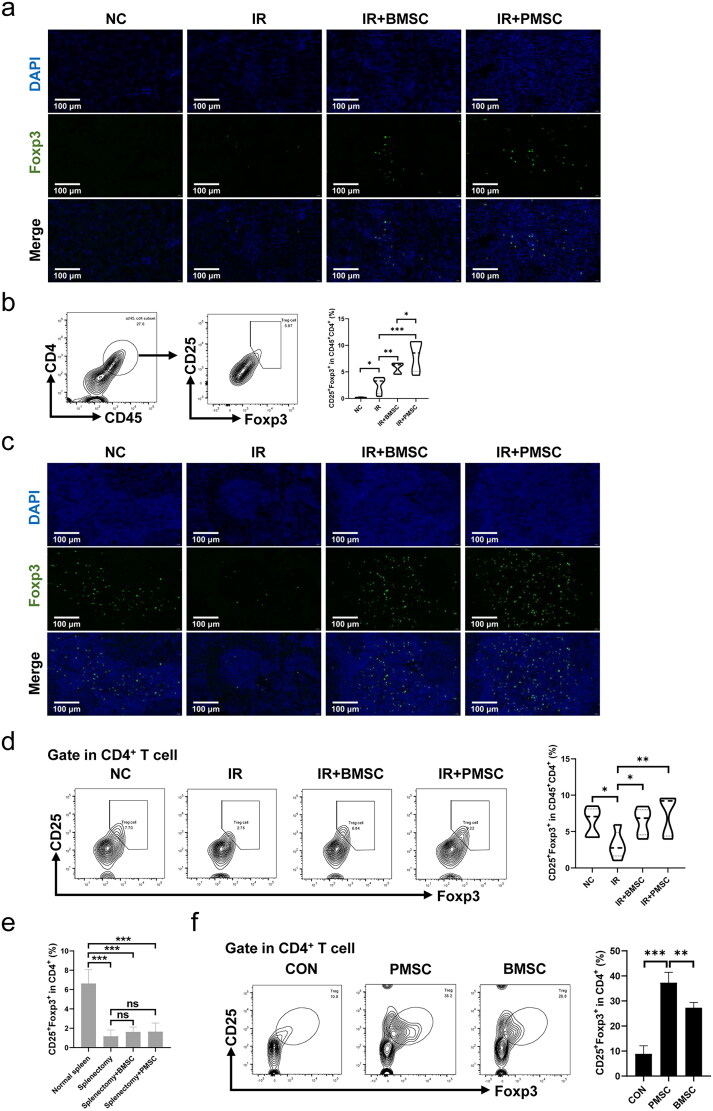
PMSCs promoted Treg increase and differentiation. (a) Immunofluorescence staining of Foxp3 (green) and DAPI (blue) in the kidney. Scale bars: 100 μm. (b) Helper T cells in the kidney were demonstrated through the expression of CD45 and CD4. Tregs were verified from helper T cells through the expression of CD25 and Foxp3. The statistical analysis of Treg proportions in helper T cells from the kidney single-cell suspension in the NC (*n* = 5), IR (*n* = 5), IR + BMSC (*n* = 5), and IR + PMSC (*n* = 5) groups. (c) Immunofluorescence staining of Foxp3 (green) and DAPI (blue) in the spleen. Scale bars: 100 μm. (d) Detection of CD45^+^CD4^+^CD25^+^Foxp3^+^Tregs from the spleen single cell suspension in the NC, IR, IR + BMSC, and IR + PMSC groups by flow cytometry. The statistical analysis of Treg proportions in helper T cells from the spleen in the NC (*n* = 5), IR (*n* = 5), IR + BMSC (*n* = 5), and IR + PMSC (*n* = 5) groups. (e) The statistical analysis of Treg proportions in CD4^+^ T cells from the kidney single-cell suspension in the normal spleen (*n* = 4), splenectomy (*n* = 4), splenectomy + BMSC (*n* = 4), and splenectomy + PMSC (*n* = 4) groups. (f) The statistical analysis of CD4^+^CD25^+^Foxp3^+^ Treg proportions in CD4^+^ T cells collected after *in vitro* culture in the CON (*n* = 3), BMSC (*n* = 3), and PMSC (*n* = 3) groups. The Kruskal–Wallis one-way analysis of variance and post hoc tests with all pairwise were used to evaluate the differences. *p < .05; **p < .01; ***p < .001.

Considering that Treg exerted a vital role in inhibiting the inflammatory reaction during IR injury, the expressions of Foxp3 in the fibrotic kidney were detected by immunofluorescence. The Foxp3^+^ Treg infiltrations in the fibrotic kidney increased after the transfer of MSCs, which was particularly obvious in the IR + PMSC group (Figure 3(a)). Meanwhile, the level of CD45^+^CD4^+^CD25^+^Foxp3^+^ Treg in the kidney was detected by flow cytometry. Compared to the NC group, there was a higher proportion of kidney Tregs (*p* < .05, Figure 3(b)) in the IR group. The transfer of either BMSCs or PMSCs significantly promoted Treg increase in the fibrotic kidney (all *p* < .01, Figure 3(b)). Moreover, the kidney Treg proportion was significantly higher in the IR + PMSC group than in the IR + BMSC group (*p* < .05, Figure 3(b)). In addition, the immunofluorescence of Foxp3 in the spleen section showed a low expression of Foxp3 in the IR group (Figure 3(c)). At the same time, the spleen Treg proportions were significantly lower in the IR group than in the NC group, IR + BMSC group and IR + PMSC group (all *p* < .05, Figure 3(d)). Moreover, either PMSC or BMSC infusion failed to increase proportion of kidney Tregs after splenectomy (all *p* > .05, Figure 3(e)). Additionally, *in vitro* co-culture of MSCs and CD4^+^CD25^–^T cells, PMSCs induced more Treg differentiation than BMSCs (*p* < .01, Figure 3(f)).

### 3.3. PMSCs ameliorated renal fibrosis through Treg

**Figure 4.**
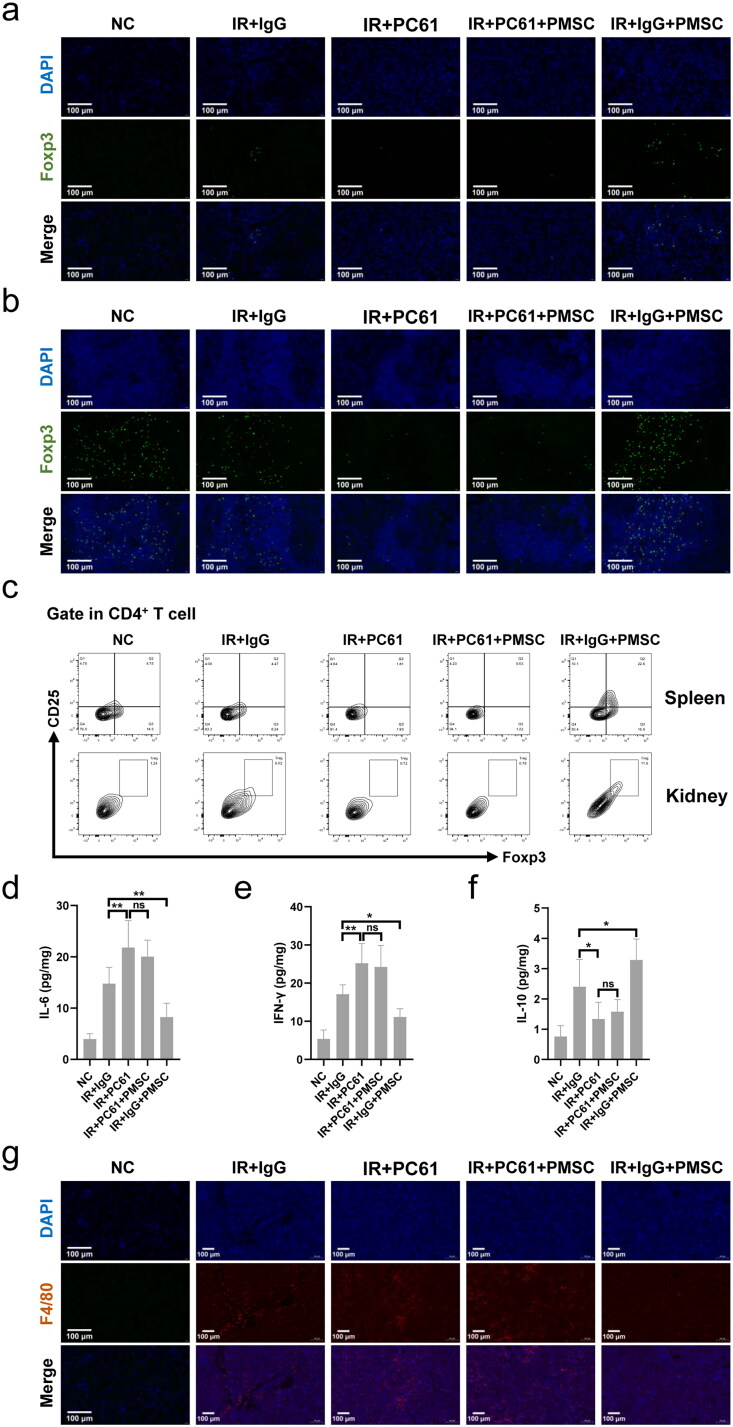
PMSCs inhibited inflammation through Tregs. The IR kidney and spleen in the NC, IR + IgG, IR + PC61, IR + PC61 + BMSC, and IR + IgG + PMSC groups were harvested on the day 2 after operation. (a, b) Immunofluorescence staining of Foxp3 (green) and DAPI (blue) in the kidney (a) and spleen (b). Scale bars: 100 μm. (c) Detection of CD45^+^CD4^+^CD25^+^Foxp3^+^Tregs from the spleen and kidney single cell suspensions using flow cytometry. The levels of IL-6 (d), IFN-γ (e), and IL-10 (f) in IR kidneys, as determined by ELISA. (g) Immunofluorescence staining of F4/80 (red) and DAPI (blue) in IR kidneys. Scale bars: 100 μm. The Kruskal–Wallis one-way analysis of variance and post hoc tests with all pairwise were used to evaluate the differences. ns, *p* > .05; *p < .05; **p < .01.

**Figure 5.**
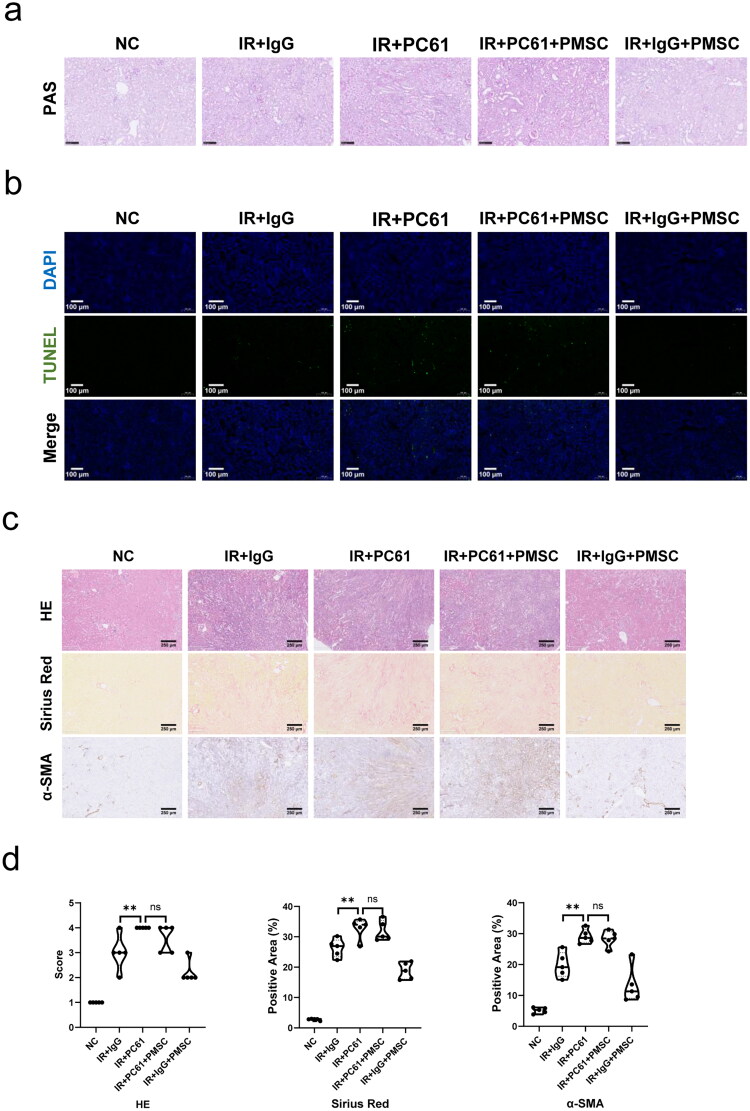
Tregs were exerted as a critical mediator during PMSC renoprotection. (a) The PAS staining was administrated to assess renal tubular injury on the day 2 after operation. Scale bars: 100 μm. (b) Immunofluorescence staining of TUNEL (green) and DAPI (blue) was administrated to assess renal tubular apoptosis on the day 2 after operation. Scale bars: 100 μm. (c, d) The HE, Sirius Red, and α-SMA staining were administrated to assess renal fibrosis on the 28th day after operation in the NC (*n* = 5), IR + IgG (*n* = 5), IR + PC61 (*n* = 5), IR + PC61 + BMSC (*n* = 5), and IR + IgG + PMSC (*n* = 5) groups. Scale bars: 250 μm. The Kruskal–Wallis one-way analysis of variance and post hoc tests with all pairwise were used to evaluate the differences. ns, *p* > .05; **p < .01.

To investigate whether Treg mainly mediated PMSC renoprotection, the PC61 was administered to deplete Treg. The immunofluorescence of Foxp3 in the kidney (Figure 4(a)) and spleen (Figure 4(b)) sections showed obvious Treg exhaustion in the IR + PC61 group and the IR + PC61 + PMSC group, which was further observed in the kidney and spleen single cell suspensions through using flow cytometry (Figure 4(c)). Subsequently, Treg immunosuppression was assessed by testing the intensity of inflammation in the kidneys. Compared with IR + IgG group, there were lower levels of proinflammatory IL-6 and IFN-γ in the IR + IgG + PMSC group (all *p* < .05, Figure 4(d–e)), with the increase of anti-inflammatory IL-10 (*p* < .05, Figure 4(f)). Meanwhile, the transfer of PMSCs increased Foxp3 but decreased F4/80 expressions (Figure 4(a,g)). Moreover, the PAS and TUNEL stainings revealed that the levels of tubular injury and apoptosis were lower following PMSC infusion (Figure 5(a,b)). However, there were opposite results occurring after Treg exhaustion (IR + IgG vs. IR + PC61, all *p* < .05, Figures 4(d–g) and 5(a,b)), with no significant differences between the IR + PC61 group and IR + PC61 + PMSC group (all *p* > .05, Figures 4(d–g) and 5(a,b)). In addition, the HE, Sirius Red, and α-SMA staining indicated that renal fibrosis was significantly more obvious in the IR + PC61 group than in the IR + IgG group (all *p* < .01, Figure 5(c,d)). Also, the renal fibrosis indexes between the IR + PC61 group and IR + PC61 + PMSC group showed no significant differences (all *p* > .05, Figure 5(c,d)).

### 3.4. mTOR pathway was associated with PMSC-induced Treg differentiation

**Figure 6.**
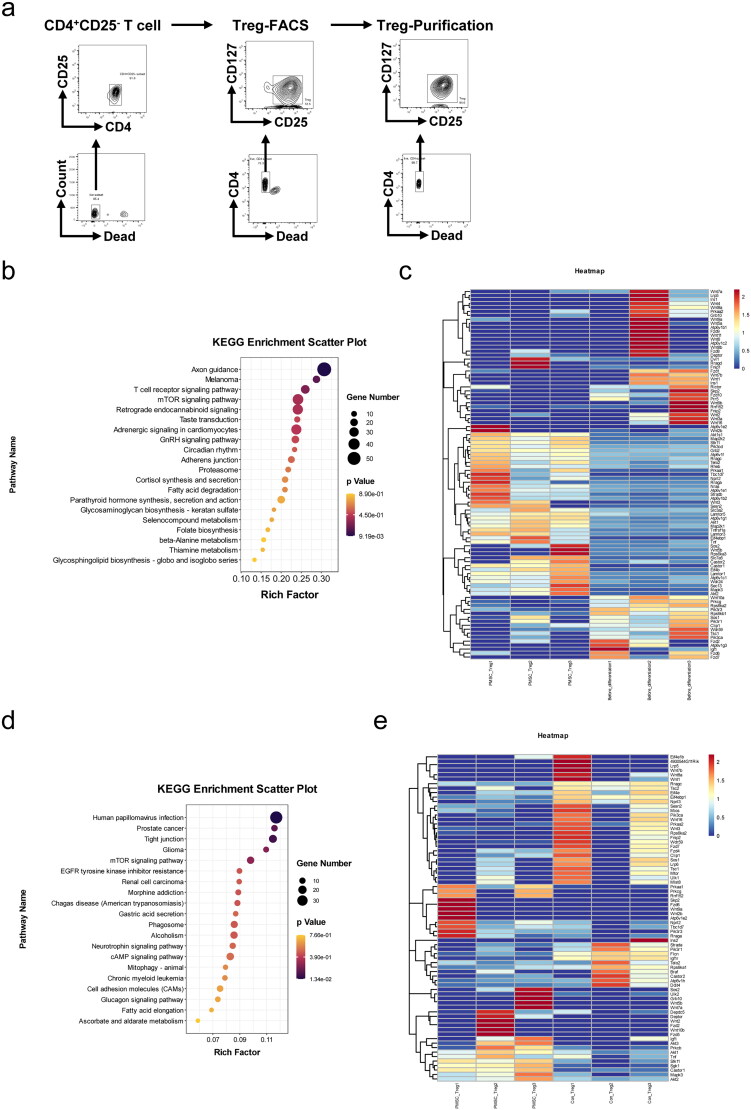
mTOR pathway participated in Treg differentiation. (a) The CD4^+^CD25^–^T cells from the spleen single cell suspension were sorted by Mouse CD4 Naïve T Cell Isolation Kit. Subsequently, the CD4^+^CD25^+^CD127^–^Tregs after 72-h stimulation *in vitro* were sorted by FACS. Eventually, the purification rate of Tregs sorted by FACS was detected by flow cytometry. (b) KEGG pathway was administered according to gene differences between PMSC-induced Tregs and undifferentiated CD4^+^CD25^–^ T cells. (c) The differential expression genes (PMSC-induced Tregs vs. CD4^+^CD25^–^ T cells) in mTOR signaling pathway. (d) KEGG pathway was administered according to gene differences between PMSC-induced Tregs and control-Tregs. (e) The differential expression genes (PMSC-induced Tregs vs. control-Tregs) in mTOR signaling pathway.

To explore the mechanism of PMSC-mediated Treg differentiation, the CD4^+^CD25^+^CD127^–^Tregs induced from CD4^+^CD25^–^ T cells were sorted by FACS, with the purification rate of Tregs occupying over 90% (Figure 6(a)). The switching mechanism at 5′ end of the RNA transcript sequencing (SMART-seq) of undifferentiated CD4^+^CD25^–^ T cells, PMSC-induced Tregs and corresponding control-Tregs were performed using bioinformatics analysis. Through analyzing the gene differences between Tregs induced by PMSC and CD4^+^CD25^–^ T cells according to SMART-seq, the Kyoto Encyclopedia of Genes and Genomes (KEGG) pathway analysis revealed that mTOR signaling pathway was associated with Treg differentiation (Figure 6(b)). Furthermore, the gene differences in the mTOR signaling pathway were presented via the heatmap (Figure 6(c)). Also, KEGG pathway analysis based on PMSC-induced Tregs vs. control-Tregs prompted that mTOR signaling pathway participated in PMSC-induced Treg differentiation (Figure 6(d)), with the heatmap of the gene differences in the mTOR signaling pathway (Figure 6(e)).

### 3.5. PMSCs regulated Treg differentiation through mTOR pathway

**Figure 7.**
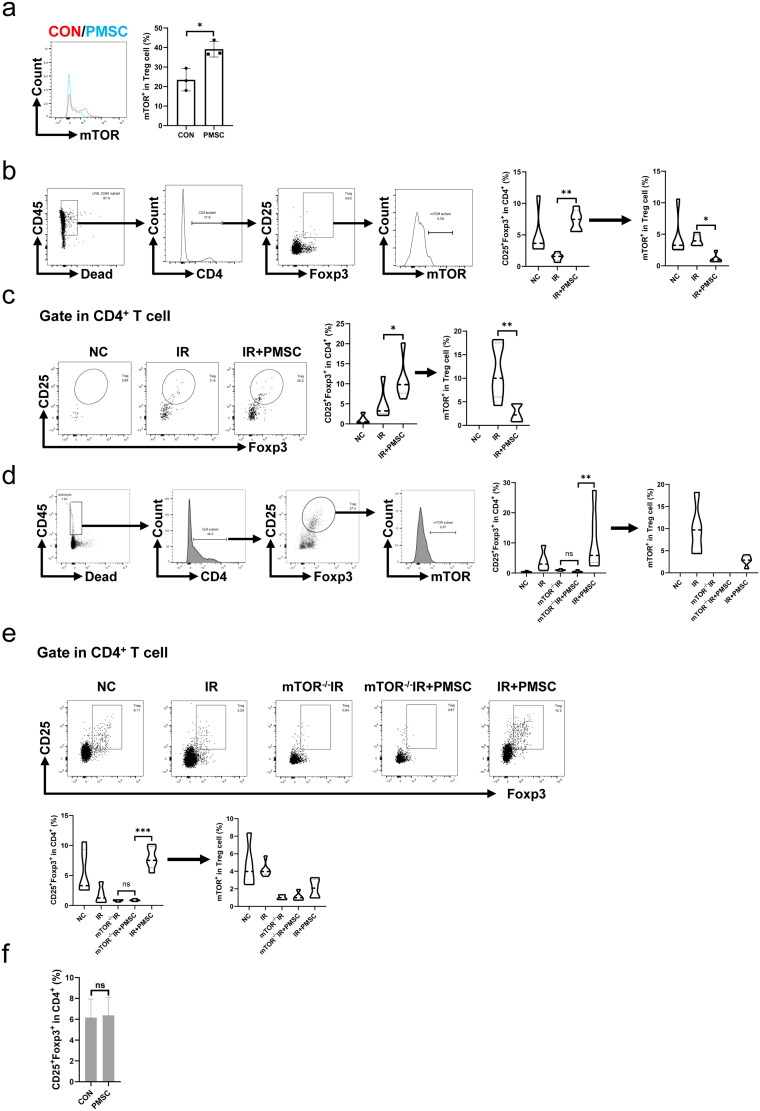
mTOR exerted as a critical mediator during PMSC promotion for Tregs. (a) The statistical analysis of mTOR phosphorylation levels in CD4^+^CD25^+^Foxp3^+^Treg collected after *in vitro* culture in the CON (*n* = 3) and PMSC (*n* = 3) groups. (b) The gating strategy to determine mTOR phosphorylation in Tregs from spleen single-cell suspension. The statistical analysis of Treg proportions in helper T cells and their mTOR phosphorylation level from Tregs in the NC (*n* = 5), IR (*n* = 5), and IR + PMSC (*n* = 5) groups. (c) Detection of CD4^+^CD25^+^Foxp3^+^Tregs from the kidney single cell suspension in the NC, IR, and IR + PMSC groups by flow cytometry. The statistical analysis of Treg proportions in helper T cells and their mTOR phosphorylation level from Tregs in the NC (*n* = 5), IR (*n* = 5), and IR + PMSC (*n* = 5) groups. (d) The gating strategy to determine mTOR phosphorylation in Tregs from kidney single cell suspension. The statistical analysis of Treg proportions in helper T cells and their mTOR phosphorylation level from Tregs in the NC (*n* = 5), IR (*n* = 5), mTOR^–/–^IR (*n* = 5), mTOR^–/–^IR + PMSC (*n* = 5), and IR + PMSC (*n* = 5) groups. (e) Detection of CD4^+^CD25^+^Foxp3^+^Tregs from the spleen single cell suspension in the NC, IR, mTOR^–/–^IR, mTOR^–/–^IR + PMSC, and IR + PMSC groups by flow cytometry. The statistical analysis of Treg proportions in helper T cells and their mTOR phosphorylation level from Tregs in the NC (*n* = 5), IR (*n* = 5), mTOR^–/–^IR (*n* = 5), mTOR^–/–^IR + PMSC (*n* = 5), and IR + PMSC (*n* = 5) groups. (f) The statistical analysis of Treg proportions in helper T cells collected after *in vitro* culture in the CON (*n* = 4) and PMSC (*n* = 4) groups. The differences for between-group comparisons were analyzed by an independent-sample *t*-test. The differences for among-group comparisons were analyzed by the Kruskal–Wallis one-way analysis of variance and post hoc tests with all pairwise. ns, *p* > .05; *p < .05; **p < .01; ***p < .001.

The levels of mTOR phosphorylation in Treg were administered through flow cytometry to explore whether the mTOR pathway was involved in PMSC-mediated Treg differentiation. First, *in vitro* co-culture of the PMSCs and CD4^+^CD25^–^ T cells showed the inhibition of mTOR phosphorylation in Treg (*p* < .05, Figure 7(a)). Subsequently, *in vivo* experiment showed that the mTOR phosphorylation of Treg from the spleen was significantly lower in the IR + PMSC group than that in the IR group, with the significantly higher Treg proportion in the IR + PMSC group and lower Treg proportion in the IR group (all *p* < .05, Figure 7(b)). Moreover, the phenomenon was also observed in the kidney (all *p* < .05, Figure 7(c)). To assess mTOR importance for PMSC-mediated Treg differentiation, tamoxifen-induced mTOR^–/–^ mice were acquired by crossing mTOR^flox/flox^ mouse with ER-Cre mouse, which was identified by western blot (Figure S3). The proportions of Tregs in both the kidney and spleen were significantly lower in the mTOR^–/–^IR + PMSC group than in the IR + PMSC group. Meanwhile, both kidney and spleen Treg proportions between the mTOR^–/–^IR group and mTOR^–/–^IR + PMSC group observed no significant differences (all *p* < .05, Figure 7(d,e)). Additionally, CD4^+^CD25^–^ T cells were sorted from mTOR^–/–^ mice. *In vitro* experiment revealed that the PMSCs failed to promote Treg differentiation following mTOR knockout (*p* > .05, Figure 7(f)).

## 4. Discussion

IR-mediated renal fibrosis with severe inflammation is a common problem, which is frequently increasing as more kidney transplantations are performed in China [2,3]. Timely inhibiting inflammatory reactions can alleviate immune damage and final renal fibrosis [1–3]. In light of MSC powerful immune regulation on inflammation, MSC infusion gradually attracts immunologist attention and is translating from animal studies to clinics a step by step [5–8]. Increasing research has shown that MSC immunoregulation was positively associated with proliferate and differentiation capacity [17–19,23]. Hence, the differences in cell proliferation and differentiation potential between PMSCs and BMSCs were administered. The growth curves showed that compared with BMSC, PMSC proliferation was more rapid. Meanwhile, the PMSCs possessed stronger adipogenic and osteogenic potential. In addition, PMSCs exerted a better effect on relieving renal fibrosis than BMSCs. These results implied that PMSCs probably were more appropriate options for treatment of IR-mediated renal fibrosis.

An increasing body of evidence indicated that MSCs exerted immunoregulation mainly by increasing Treg infiltration in the kidney, which was vital to inhibit the degree of inflammation in IR kidney [24–26]. Our data showed that compared with BMSCs, PMSCs more effectively promoted Treg increase and differentiation. Treg exhaustion experiment further demonstrated that Tregs exerted an important effect on increasing anti-inflammatory IL-10 level and suppressing proinflammatory IL-6, IFN-γ, and F4/80 expressions. After Treg depletion, the PMSCs failed to inhibit renal inflammation and relieve renal fibrosis. These results indicated that the PMSCs ameliorated renal fibrosis mainly via Treg immunosuppression. In addition, our group found that spleen Treg proportions in the IR group were lower than those in the NC group. However, either PMSC or BMSC infusion after IR promoted spleen Treg return to normal levels. Several studies have shown that MSCs mainly resided in the lungs, partially reached the spleen, and rarely infiltrated into the kidney after intravenous infusion [27–29], which implied that the increase of Treg infiltration in IR kidney probably attributed to PMSC promotion of Treg differentiation in the spleen. Indeed, the transfer of both PMSCs and BMSCs failed to increase kidney Treg proportion after splenectomy.

To explore the mechanism of PMSC-mediated Treg differentiation, the Tregs stimulated by the PMSCs *in vitro* were administered by SMART-seq. The results of SMART-seq showed that PMSCs promoted Treg differentiation via the mTOR pathway. Proverbially, the mTOR is the main regulator of the differentiation of Naïve CD4^+^ T cells, which were Treg’s common precursor cells. A growing number of researches have shown that under hypoxia conditions, mTOR phosphorylation of Naïve CD4^+^ T cells are easily promoted to mediate hypoxia-inducible factor-1α (HIF-1α), which further increases phosphorylation of signal transducer and activator of transcription (STAT)-3 and finally downregulated the transcription factor forkhead box protein P3 (Foxp3) expression. However, MSCs could interfere with the mTOR phosphorylation during the differentiation of Naïve CD4^+^ T cells, thus leading to HIF-1α downregulation, STAT5 phosphorylation increase, Foxp3 expression, and Treg production [9,30–32]. Therefore, *in vivo* and *in vitro* experiments, detecting the mTOR phosphorylation in Treg revealed that PMSC promoted an increase of Tregs and their mTOR inhibition. However, the PMSCs failed to promote Treg increase and differentiation after mTOR knockout.

The present study innovatively showed that PMSCs, as a new type of MSCs, more effectively promoted Treg increase and alleviated renal fibrosis. Importantly, combined with existing literatures [27–32], these findings in this study provide a hypothesis that PMSCs may circulate to the spleen, subsequently inhibiting mTOR phosphorylation of Naïve CD4^+^ T cells, further promoting Naïve CD4^+^ T cells to differentiate into Tregs, thereby leading to the recruitment of Tregs in the kidney, ultimately inhibiting inflammatory process and effectively alleviating renal fibrosis. However, in the future, the main mediator of PMSC inhibition of mTOR and promotion of Tregs should be further explored. In addition, it was necessary to explore the growth of implanted PMSCs, PMSC survival time *in vivo* and the biosafety problem.

## Data Availability

Data for the information presented in the manuscript can be directed to the corresponding authors.
